# Bilateral teleoperation with object-adaptive mapping

**DOI:** 10.1007/s40747-021-00546-z

**Published:** 2021-10-15

**Authors:** Xiao Gao, João Silvério, Sylvain Calinon, Miao Li, Xiaohui Xiao

**Affiliations:** 1 Hubei Key Laboratory of Waterjet Theory and New Technology, Wuhan University, Wuhan, China; 2 Idiap Research Institute, Martigny, Switzerland; 3 National Key Laboratory of Human Factors Engineering, China Astronauts Research and Training Center, Beijing, China

**Keywords:** Bilateral teleoperation, Motion mapping, Invertible mapping, Impedance control

## Abstract

Task space mapping approaches for bilateral teleoperation, namely object-centered ones, have yielded the most promising results. In this paper, we propose an invertible mapping approach to realize teleoperation through online motion mapping by taking into account the locations of objects or tools in manipulation skills. It is applied to bilateral teleoperation, with the goal of handling different object/tool/landmark locations in the user and robot workspaces while the remote objects are moving online. The proposed approach can generate trajectories in an online manner to adapt to moving objects, where impedance controllers allow the user to exploit the haptic feedback to teleoperate the robot. Teleoperation experiments of pick-and-place tasks and valve turning tasks are carried out with two 7-axis torque-controlled Panda robots. Our approach shows higher efficiency and adaptability compared with traditional mappings.

## Introduction

Teleoperation is widely used in hazardous environments, such as outer space [1], deep sea [2], and nuclear industry. Since its inception [3], the field has witnessed great progress, owing to novel control paradigms (e.g. torque control), new haptic interfaces and the increase in sensor modalities and perception technologies. The advent of the latter, while allowing to operate in more and more complex environments, has given rise to new problems. Traditional direct control architectures rely on the user’s skills to control all slave motion, which leads to big mental workload. Supervisory control is developed to reduce workload, as the user can teleoperate the slave robot by high-level commands, rather than low-level motions. However, it it complicated to apply strong autonomy on the slave robot in complex environments. Shared control locates between direct control and supervisory control. The slave robot is controlled by both direct control and remote autonomy.

**Fig. 1:**
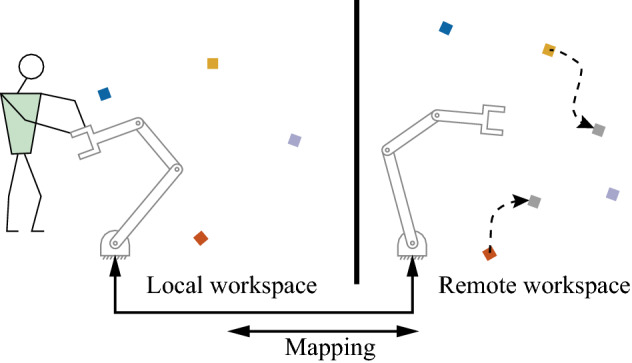
A schematic of bilateral teleoperation for multiple objects. Left: Local workspace. Right: remote workspace. Colored squares represent the objects to be manipulated. Our approach builds an online mapping between the local and remote workspace based on object positions

Various teleoperation modalities exist, with limitations appearing as the complexity of the task context grows. For example, a user needs to teleoperate an underwater remote operated vehicle (ROV) to pick some objects or turn several valves. Based on 2D video streaming, the user controls the ROV by a haptic device to approach one and pick it. Then press a button to suspend the teleoperation and relocate the haptic device for the next subtask. If the objects to be manipulated are pushed by the water flow, the user must adjust the ROV to a new location and perform the task carefully. Specifically, as shown in Fig. 1, when the *remote* robot needs to operate objects which are placed differently from the *local* workspace, mapping the motions of the two robots is not straightforward.

To address the problems of discrepancies between local and remote spaces and teleoperation for moving objects, we consider mappings that locally reshape the robot task space in a way that ensures successful manipulation on the remote side. Particularly, we consider the location of objects on both local and remote sides and propose a locally linear mapping approach to build a mapping between the two operational spaces. Thus, the user can focus on only the task on his/her side by kinesthetically moving the local robot end-effector. The mapping will generate the desired pose online and the remote side will perform the same task with different location setups, which makes the teleoperation more intuitive and efficient. And this mapping is an object-adaptive one, which means it can be updated online and adapt to moving objects. To validate the approach, we propose robot experiments in three scenarios where bilateral teleoperation is exploited. Experiments for collect moving objects are designed to show the online adaptability of the mapping, along with groups of trials to highlight the efficiency in comparison to traditional teleoperation mappings.

This paper is organized as follows. We discuss previous research in “Related work”. “Object-adaptive mapping” describes our object-adaptive mapping algorithm. “Experiments” shows the real robot experiments, including pick-and-place ball tasks, collecting moving objects and valves-turning tasks. We discuss the limitations in “Discussion”. “Conclusion” concludes the paper.

## Related work

Bilateral teleoperation allows the user to give motion commands to the slave by guiding a local joystick or robot, while the user can feel the slave interaction force. In this paper, we focus on how to map robot actions between the master and slave sides in Fig. 1.

Mimicking the joint configurations of the master is likely the most straightforward way of teleoperation [4–7]. Limitations of this approach are evident: when the device being used on the master side has a different kinematic structure than the robot being controlled, the mapping is not intuitive (e.g. from linear motions of a joystick to rotations of revolute joints). This typically results in increased cognitive load for the user, which in turn leads to suboptimal performance [8, 9]. Successful teleoperation in these scenarios often relies strongly on the expertise of experienced users, who endured time-consuming training [6, 8]. Finding appropriate subspaces to represent the joint mappings has received attention, not only in the context of manipulators but also in grasping [7].

Reformulating joint space approaches into the robot task space has been shown to improve performance and user experience [4, 8, 9]. Indeed, most modern approaches rely on this paradigm. Starting from the original work of Goertz [3], where the 6-DOF of the *master handle* are mechanically linked to those of the *slave hand*, to more recent approaches considering *virtual fixtures* [10–13], task space representations have become ubiquitous in teleoperation. Among these approaches, the object-centered ones are typically the most successful. Abi-Faraj et al. [14] rely on a camera to autonomously control some of the *slave* robot’s degrees of freedom, given detected object poses in a shared control scenario.

However, common approaches require clutching phases when the robots in the local and remote sides are disconnected. The goal is to adjust the offsets between two robots, as the two sides might be different. In general, several clutching phases exist for teleoperating one objects. In teleoperation for multiple objects, it will be too time-consuming.

Semi-autonomous teleoperation has been widely used to reduce mental workload. Learning from demonstration approaches are introduced to reproduce skills given human motion examples [15]. In [16], a framework is presented with task-parametrized representation in a mixed teleoperation setting, which can resolve differences between local and remote environment configurations to improve transparency in a subtask. However, teleoperating the remote robot to manipulate several objects is still discontinuous due to the clutching phases, which leads to low transparency and efficiency. For complex scenarios where the decision-making of commands is very difficult or changed rapidly, we may have to rely on ourselves to send realtime commands. In our previous work [17], two motion mapping methods were proposed for continuously bilateral teleoperation. However, both methods cannot adapt to moving objects online, as they take long time for re-training. Thus it is impossible for the user to teleoperate a moving object.

With respect to the aforementioned, our approach ensures an invertible mapping between the two workspaces. It can bring better transparency and efficiency in bilateral teleoperation for multiple objects. It also adapts to scenarios of online moving objects.

## Object-adaptive mapping

Given the specified one-to-one corresponding points, with the 3D position of objects $\varvec{X}=\{{\varvec{x}}_i\}_{i=1}^N$ and $\varvec{Y}=\{{\varvec{y}}_i\}_{i=1}^N (N\ge 4)$ in the local and remote workspaces respectively, our goal is to build a realtime bijective mapping $\varPhi$ between $\varvec{X}$ and $\varvec{Y}$, by taking into account typical paths formed when moving from one location to another. We exploit here the prior knowledge that a motion from one location to another will mainly form a straight line in Cartesian space, for an environment without obstacle and for regions in the workspace that are easily reachable.

One potentially promising solution is to use a diffeomorphic mapping to minimize the distance between $\varPhi (\varvec{X})$ and $\varvec{Y}$. Perrin et al. [18] adopted a locally weighted translation with many Gaussian Radial Basis Functions (RBF) by iteration method, and received a bijective and differentiable mapping function. However, the time cost both for learning and backward evaluation is high, which may cause long delays, lowering the *transparency* in bilateral teleoperation.

We propose a locally linear mapping algorithm based on the locally coordinate representation, which is much faster for realtime teleoperation and can meet the requirement of moving in straight lines between points of interest. The mapping is described in Cartesian space for the poses of robot end-effectors, so that the local robot motion can be mapped to and from the remote side.

**Fig. 2:**
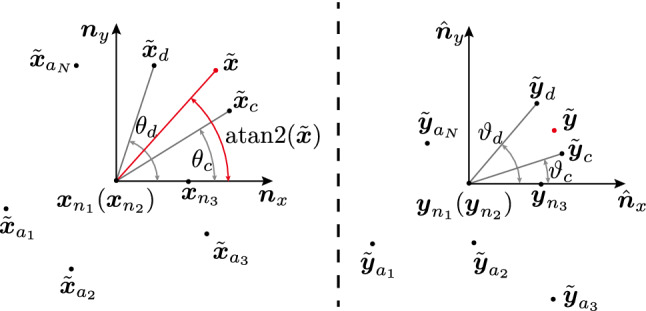
Left: Left task space. Right: Right task space. Points with projection are sorted by angles of atan2 function. And the points of $\tilde{{\varvec{x}}}_d$ and $\tilde{{\varvec{x}}}_c$ are found with $\theta _c \le \text {atan2}(\tilde{{\varvec{x}}})<\theta _d$. Thus, ${\varvec{x}}$ is represented by the four points of ${\varvec{x}}_{n1}, {\varvec{x}}_{n2},{\varvec{x}}_{c},{\varvec{x}}_{d}$. And the mapping point ${\varvec{y}}$ is generated in the same region

### Locally linear mapping

Assumed that there is an initial point ${\varvec{x}}$ of the end-effector on the local task space, initially we choose the nearest point $n_1=\underset{i \in [1,\ldots ,N]}{\arg \min }(|| \{{\varvec{x}}_i\}_{i=1}^N- {\varvec{x}} ||)$ as initial mapping center point. The algorithm is described as follows:

1. Check the nearest distance between ${\varvec{x}}$ and $\{{\varvec{x}}_i\}_{i=1}^N$. If it is less than a threshold, update $n_1$ as that point index.
2. Start a loop to choose $n_2 \in N$ as a second point for the ordered basis. Then select $n_3 \in N (n_3 \ne n_1,n_2)$
3. Build projection function. First, we build a Cartesian coordinate system with ${\varvec{x}}_{n_1}$ for center and ${\varvec{x}}_{n_2} - {\varvec{x}}_{n_1}$ for ${\varvec{z}}$ axis, and get ${\varvec{x}}$ axis by making a projection of ${\varvec{x}}_{n_3} - {\varvec{x}}_{n_1}$ on the vertical plane around the ${\varvec{z}}$ axis.
4. Get the 2D projection points as $\tilde{{\varvec{X}}},\tilde{{\varvec{x}}},\tilde{{\varvec{Y}}}$ from new the xy plane (see Fig. 2).
5. Calculate the angle lists. We sort the points by atan2 function and get the index lists ${\varvec{a}} , {\varvec{b}}$ and angle lists $\varvec{\theta }, \varvec{\vartheta }$. ${\varvec{a}}$ should be equal to ${\varvec{b}}$, otherwise continue the *For* loop.
6. Decide which region $\tilde{{\varvec{x}}}$ is in. As the return value of atan2 function is in $(-\pi , \pi ]$, we can clearly know that ${{\,\mathrm{atan2}\,}}({\tilde{{\varvec{x}}}})$ is between $\theta _{c}$ and $\theta _{d}$, shown in Fig. 2.
7. Choose the best ${\varvec{n}}_2$. We define a distortion function: $\varvec{\rho }= \sum \exp (| \ln (\varDelta \varvec{\theta }/\varDelta \varvec{\vartheta }) |)$ and assign to ${\varvec{n}}_2$ to get the minimum value of $\varvec{\rho }$.
8. Compute the output. We receive the ordered basis $\varvec{\alpha }=[ {\varvec{x}}_{n2}- {\varvec{x}}_{n1} ,{\varvec{x}}_{n3}- {\varvec{x}}_{n1},{\varvec{x}}_{n4}- {\varvec{x}}_{n1} ]$ for ${\varvec{x}}$ and $\varvec{\beta }= [ {\varvec{y}}_{n2}- {\varvec{y}}_{n1} ,{\varvec{y}}_{n3}- {\varvec{y}}_{n1},{\varvec{y}}_{n4}- {\varvec{y}}_{n1} ]$ for ${\varvec{y}}$. The representation of ${\varvec{x}}$ with respect of $\varvec{\alpha }$ is $\varvec{\lambda }= \varvec{\alpha }^{-1}({\varvec{x}} - {\varvec{x}}_{n_1})$. ${\varvec{x}}$ and ${\varvec{y}}$ are set as the same coordinates under the corresponding ordered basis. The output of the algorithm is ${\varvec{y}} = \varvec{\beta }\varvec{\lambda }+{\varvec{y}}_{n_1}$, $\varvec{\dot{y}} = \varvec{\beta }\varvec{\alpha }^{-1}\varvec{\dot{x}}$.

**Fig. 3:**
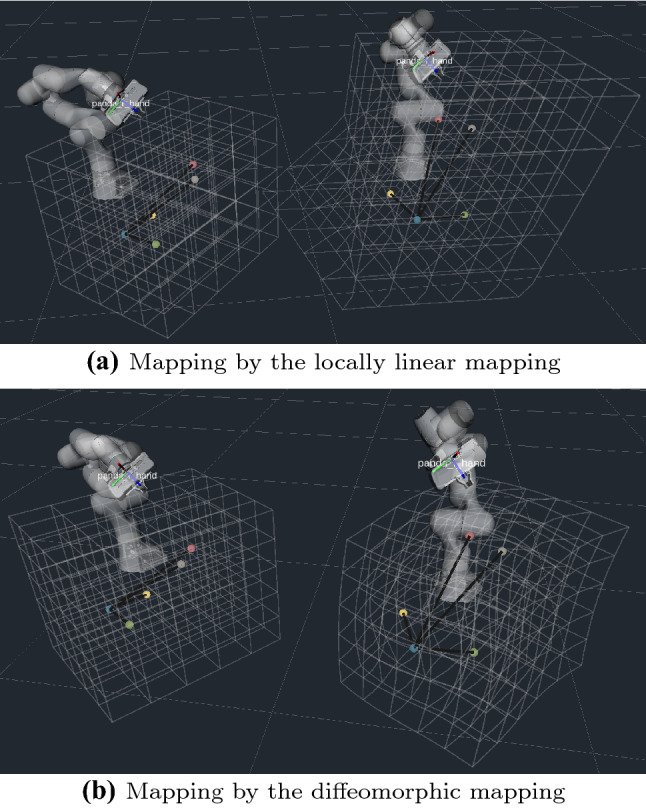
Reshaped robot task spaces in ROS Rviz visualizer. Colored points refer to the objects to be manipulated. Left: Local workspace with a Panda robot as master. Right: Remote workspace with a robot as slave

**Table 1:** Comparison of simulation results

	Locally linear	Diffeomorphism
Learning time	0	558 ms
Forward evaluation: time cost of $\varPhi ({\varvec{X}})$	1 ms	3 ms
Backward evaluation: time cost of $\varPhi ^{-1}({\varvec{Y}})$	1 ms	25 ms
Mean error: dis($\varPhi ({\varvec{X}})-{\varvec{Y}}$)	0	2.7 mm

Notice that there is input–output symmetry to guarantee bilateral teleoperation. The algorithm decides which region ${\varvec{x}}$ is in and chooses nearby vectors for the ordered basis and generates a corresponding point with the same coordinates on the other side. The main idea of the algorithm is to select an ordered basis as representation of points. Coordinates are transferred to the other side to rebuild the points. Thus, a linear mapping is built in each region.

Algorithm 1 shows the pseudocode of the proposed approach.
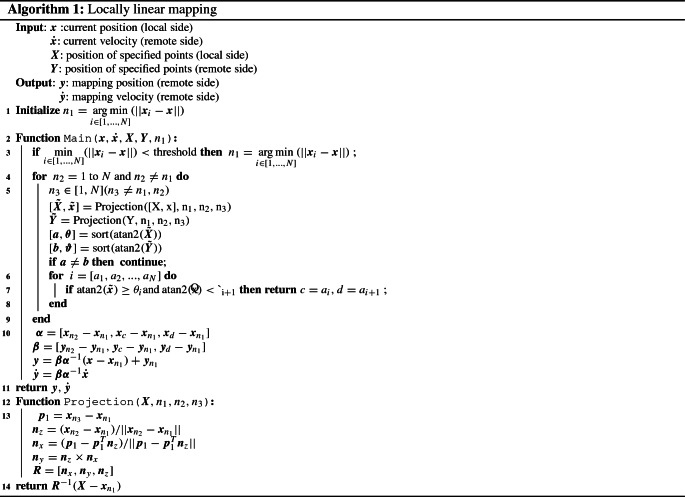


### Comparison in simulation

In this part, we compare the proposed locally linear mapping and a diffeomorphic mapping [18] in simulation, with five different points set in both robots’ task spaces. For diffeomorphic mapping, one point is chosen as the center point and connected with other points by straight lines. 200 evenly spaced samples on these lines are set for generating the mapping. We adopt the algorithm in [18] for the diffeomorphic mapping.

Simulation results are shown in Fig. 3. On the left side, the equal-spaced grid lines are plotted to show the local worspace. The remote workspace is deformed by the mapping on the right side.

The space of Fig. 3a is divided by regions with the order of angle in Algorithm 1. Then in each region a linear mapping is applied. The diffeomorphic mapping transforms the sample points on the gray lines. And it shows a smooth distortion in Fig. 3b. In Table 1, the characteristics of the two mapping methods are compared. In this case, the diffeomorphic mapping would cost 558 ms for learning the mapping by iterative approximation (iteration number = 200), with a mean error of 2.7 mm. And in the backward evaluation, nonlinear equations should be solved to calculate the corresponding point from the right side to the left side of Fig. 3b, which takes 25 ms. For the locally linear mapping, there is no need for learning and have a fast computation time without error, which is better for realtime control (we further discuss these aspects in Sect. 5).

**Fig. 4:**
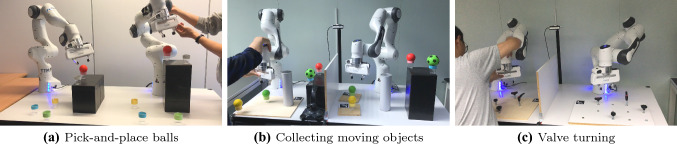
Three scenarios of teleoperation experiments. **(a)** Experimental setup of pick-and-place balls. **(b)** Collecting four balls into a container. **(c)** Turning valves by $90^{\circ }$. The user side of each subfigure refers to local workspace for human guidance, and the other side for remote robot manipulation. The goal is to manipulate the remote objects in sequence by teleoperation. A vertical board prevents the user from relying on visual feedback to complete the task, emulating the realistic teleoperation of a remote robot

**Fig. 5:**
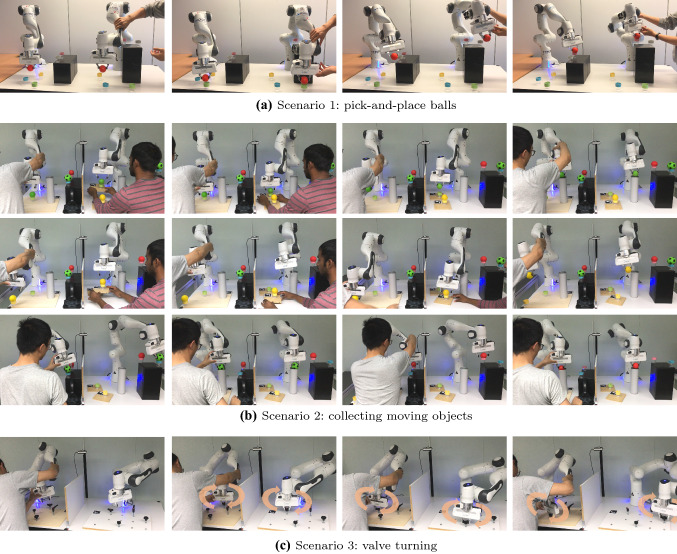
Snapshots of teleoperation experiments by the locally linear mapping in three scenarios. **(a)** The user guides the right robot to pick the red ball, pass by the gray cup and place it on the pink cup. **(b)** The user (left side) guides the robot to collect four balls into the cylindrical container, while the balls on the remote side may move arbitrarily. **(c)** The user teleoperates the robot to turn the valves in sequence

**Fig. 6:**
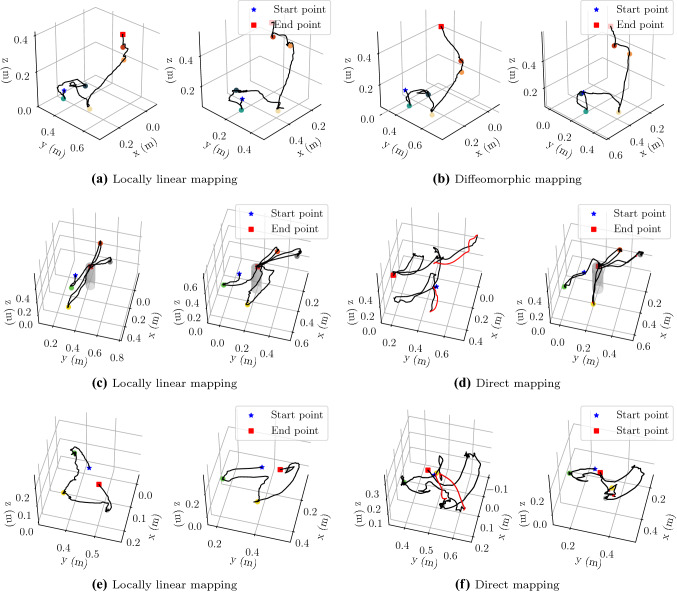
Robot trajectories. *First to third rows*: the corresponding three scenarios. *1st and 3rd columns*: the local workspace. *2nd and 4th columns*: the remote workspace. **b**–**d**: baselines for our method. Robot trajectories are displayed in black. And the red ones in **d** and **f** refer to the trajectories when the teleoperation is suspended due to the direct mapping. Colored points refers to the objects to be manipulated

## Experiments

To validate the proposed locally linear mapping algorithm, three sets of experiments were conducted with bilateral teleoperation, including pick-and-place static objects, collecting moving objects and turning valves, as shown in Fig. 4. We consider the direct position mapping [19] and the diffeomorphic mapping [18] as baselines to evaluate our method.

### Controller

The two robots used in the experiments are torque-controlled, where impedance controllers are adopted to allow the operator to guide the local robot and teleoperate the remote one in Fig. 4. The control law is designed for both robots as:

1 $$\begin{aligned} {\varvec{\tau }} = {\varvec{M}} ({\varvec{q}}) {\ddot{\varvec{q}}} + {\varvec{g}}({\varvec{q}}) + {\varvec{c}}({\varvec{q}}, {\dot{\varvec{q}}}) +{\varvec{J}}({\varvec{q}})^T {\varvec{f}}, \end{aligned}$$

with

2 $$\begin{aligned} {\varvec{f}} =\begin{bmatrix}{\varvec{k}}_p (\varvec{x}_d - \varvec{x}) +{\varvec{k}}_v( {\dot{\varvec{x}}}_d - {\dot{\varvec{x}}}) \\ {\varvec{k}}_{pr} \text {log}({\varvec{R}}_d {\varvec{R}}^T) +{\varvec{k}}_{vr}( \varvec{\omega }_d - {\varvec{ \omega }}) \end{bmatrix}, \end{aligned}$$

where ${\varvec{q}} \in \mathbb {R}^7$ denotes joint position, ${\varvec{M}}({\varvec{q}}) \in \mathbb {R}^{7 \times 7}$ is the mass matrix, ${\varvec{c}}({\varvec{q}}, {\dot{\varvec{q}}}) \in \mathbb {R}^7$ is the Coriolis and centrifugal torque ${\varvec{J}}({\varvec{q}}) \in \mathbb {R}^{6 \times 7}$ is the Jacobian matrix, and ${\varvec{\tau }}$, ${\varvec{g}}({\varvec{q}}) \in \mathbb {R}^7$ are respectively control and gravitational joint torques. ${\varvec{f}} \in \mathbb {R}^{6}$ is the output of the impedance controller. $\varvec{x}, {\dot{\varvec{x}}} \in \mathbb {R}^3$ are the Cartesian position and velocity. ${\varvec{R}}$ is the $3 \times 3$ orientation matrix and ${\varvec{ \omega }} \in \mathbb {R}^{6}$ the angular velocity of the end-effector. Here the logarithmic function of a rotation matrix is adopted for the difference between two rotation matrices. $(.)_d$ denotes the desired value, and ${\varvec{k}}_{(.)}$ represents stiffness and damping gains (for position and orientation) of the Cartesian impedance controller.

To perform the teteoperation task with different point locations on two sides, we use the mapping function $\varPhi$ in Algorithm 1 of Sect. 3 to generate motion online. The desired value (reference trajectories) in Eq. 2 is set as :

3a $$\begin{aligned}&\varvec{x}_d^r, \varvec{\dot{ x}}_d^r = \varPhi (\varvec{x}^l, \varvec{\dot{ x}}^l), \quad \quad {\varvec{R}}_d^r ={\varvec{R}}^l, \quad {\varvec{ \omega }}_d^r = {\varvec{ \omega }}^l , \end{aligned}$$

3b $$\begin{aligned}&\varvec{x}_d^l, \varvec{\dot{ x}}_d^l = \varPhi ^{-1}(\varvec{x}^r, \varvec{\dot{ x}}^r), \,\,\,\, {\varvec{R}}_d^l={\varvec{R}}^r , \; \;\; {\varvec{ \omega }}_d^l = {\varvec{ \omega }}^r , \end{aligned}$$

where $(.)^r$ and $(.)^l$ mean the state of the right robot and the left robot respectively. Therefore, each robot is controlled to track a reference position, generated by the mapping function that takes the other robot’s position as input. In this case, only position and velocity are considered in the mapping, while desired orientation and angular velocity are copied from the other robot. Gain matrices are set as ${\varvec{k}}_p = 300 {\varvec{I}}_{3\times 3},{\varvec{k}}_v = 10 {\varvec{I}}_{3\times 3}, \varvec{k}_{pr} = 15 {\varvec{I}}_{3\times 3},{\varvec{k}}_{vr} = 2 {\varvec{I}}_{3\times 3}$, where ${\varvec{I}}_{3\times 3}$ is the three-order identity matrix. This implementation therefore corresponds to position-position (as opposed to e.g. position-force) architecture where both sides run an impedance controller to track a reference trajectory which is generated by the motion mapping algorithm.

### Robot experiments

The three sets of experiments are shown in Fig. 4a–c. In each scenario, there are several corresponding objects to be manipulated in the local and remote sides, with different locations for each pair of objects. As illustrated in Fig. 4a, there are five colored cups on both sides to represent the objects of interest, and a red ball to be grasped and placed. The grasping position of the red ball on each cup is previously recorded. In the second and third scenarios, a camera (RealSense D435) is mounted on the table to locate the boards, and consequently the objects, by using Aruco makers. The transformations between the balls and the markers, as well as between the camera and the robots, are calibrated in advance. In scenario 1, we adopt the locally linear mapping and the diffeomorphic mapping for the teleoperation mapping. In scenario 2 and 3, we adopt the locally linear mapping and the direct position mapping [19], which only copies the relative pose of the local robot and sends to the remote robot. The vertical board are removed when using the direct position mapping, so that the user can watch the remote workspace directly, which emulates the realistic teleoperation by video streaming feedback.

A video^1^ accompanying this paper shows the results of the experiments. Figure 5a–c show the snapshots in the three scenarios by the locally linear mapping. Figure 3 shows how the robot operational spaces were distorted by the locally linear mapping and the diffeomorphic mapping. Robot trajectories of the end-effector on both sides are displayed in Fig. 6. From the results, all methods fully completed the task.

In the execution of scenario 1, the locally linear mapping and diffeomorphic mapping methods bring similar trajectories. Both of them can deal with the discrepancies of location of objects on two sides. And a continuous teleoperation for multiple objects is achieved. In Fig. 6d,f, the trajectories in red mean that the user suspended the teleoperation and adjusted the local robot to a new location, while the teleoperation by our locally linear mapping did not require this phase. The trajectories (Fig. 6a,c,e) are continuous for manipulating multiple objects.

Besides, the locally linear mapping can adapt to online movement of remote objects. In Fig. 5b for scenario 2, the human on the right side was applying random movement to a ball, while the user can still teleoperate the robot to collect the moving ball. Note that the diffeomorphic mapping required re-training for moving objects. Thus, in case the points of interest change locations during the execution, our approach can generate new mappings online without increasing delays.

**Fig. 7:**
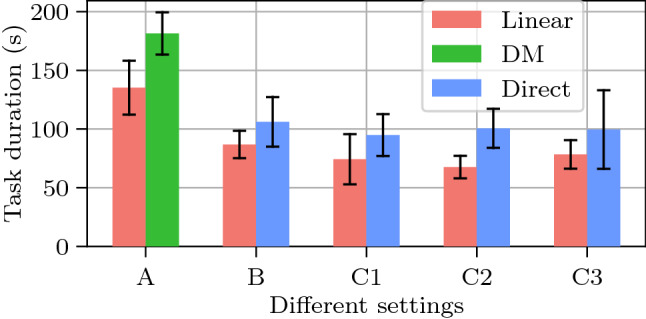
Comparisons of task duration by different methods. *A*: scenario 1. *B*: scenario 2. *C1-C3*: scenario 3 with three different position settings of valves. Each setting includes 20 trials. The error bar shows the standard deviation

In Fig. 7, we compare the task duration of the three mapping methods in five different settings. The results show that the locally linear mapping takes less time than the others, which means better efficiency. As our method does not suspend the teleoperation, the user can focus on the local side and complete subtasks continuously. Mostly the direct mapping has bigger standard deviation. One possible reason is that sometimes the user needs more or clutching phases due to physical constraints.

## Discussion

We compared our proposed mapping with the one from [18] and the direct mapping [19]. In our chosen setup, the objects of interest were in different locations between the workspaces of the two robots and some of them were moving online. The task was successfully executed by all three approaches. However, our approach took less time to accomplish the task and less time cost of realtime computation, which makes it more suitable for bilateral teleoperation as they are directly linked to both teleoperation efficiency and transparency, where computational times should be kept as low as possible due to the constraint of operating in real time.

Some limitations are, nonetheless, worth mentioning. First, our approach requires that objects are placed in the same order in both workspaces. When this is not verified, the mapping could result in distortions that map otherwise reachable points outside the workspace of the robots. Second, our mapping is continuous but not differentiable at the plane switch (see Sect. 3). The diffeomorphic approach [17, 18], contrarily, can build functions with higher orders of continuity and only distort the neighbouring space of the points. However, this comes at the cost of high training and evaluation times, which are often prohibitive in teleoperation, especially in the bilateral case. Finally, it is worth mentioning that our approach shows more promise for dynamic scenarios, where objects are moving in either one of the workspaces. This is because it does not require learning time (see Table 1), hence the mapping can be quickly updated as opposed to other alternatives like [17] and [18], where recomputation takes a considerable amount of learning time.

## Conclusion

In this paper, we propose a locally linear mapping algorithm to map points between two robot workspaces, in bilateral teleoperation scenarios. Considering objects in different locations and the requirement of adaptability to moving objects, this algorithm can distort the space in different regions and generate an object-centered mapping. This mapping can also be updated online when the objects are moving. We validated the algorithm with experiments of three scenarios, and contrasted it with a diffeomorphic mapping and the direct mapping. While we showed that both algorithms allow for successfully completing the task, our approach stands out due to lower computational time and faster task completion, which are reflected in higher teleoperation efficiency. In future work, we plan to use more elaborated visual feedback to incorporate moving objects, investigate the consideration of obstacles in the mapping [20] and study techniques for motion prediction that can facilitate the teleoperation when objects move.

## Footnotes

1 The video is also available online at https://youtu.be/iS9bykV5jJM.

## References

[1] Lii NY, Chen Z, Pleintinger B, Borst CH, Hirzinger G, Schiele A (2010) Toward understanding the effects of visual- and force-feedback on robotic hand grasping performance for space teleoperation. In: Proc. IEEE/RSJ Int. Conf. Intell. Robot. Syst. (IROS), pp 3745–3752

[2] Murphy RR, Dreger KL, Newsome S, Rodocker J, Steimle E, Kimura T, Makabe K, Matsuno F, Tadokoro S, Kon K (2011) Use of remotely operated marine vehicles at minamisanriku and rikuzentakata japan for disaster recovery. In: IEEE International Symposium on Safety, Security, and Rescue Robotics, pp 19–25

[3] Goertz RC (1949) Master-slave manipulator. Tech. rep., Argonne National Laboratory, USA

[4] Girmscheid G, Moser S (2001). Fully automated shotcrete robot for rock support. Comput Aided Civil Infrastruct Eng.

[5] Romano JM, Webster RJ, Okamura AM (2007) Teleoperation of steerable needles. In: Proc. IEEE Int. Conf. Robot. Autom. (ICRA), pp 934–939

[6] Marturi N, Rastegarpanah A, Takahashi C, Adjigble M, Stolkin R, Zurek S, Kopicki M, Talha M, Kuo JA, Bekiroglu Y (2016) Towards advanced robotic manipulation for nuclear decommissioning: a pilot study on tele-operation and autonomy. In: Int. Conf. on Robot. and Autom. for Humanitarian Applications (RAHA), pp 1–8

[7] Meeker C, Rasmussen T, Ciocarlie M (2018) Intuitive hand teleoperation by novice operators using a continuous teleoperation subspace. In: Proc. IEEE Int. Conf. Robot. Autom. (ICRA), pp 5821–5827

[8] Mowe CE, Merkt W, Davies A, Vijayakumar S (2019) Comparing alternate modes of teleoperation for constrained tasks. In: IEEE Int. Conf. on Autom. Science and Engineering (CASE), pp 1497–1504

[9] Majewicz A, Okamura AM (2013) Cartesian and joint space teleoperation for nonholonomic steerable needles. In: Proc. World Haptics Conference (WHC), pp 395–400

[10] Abi-Farraj F, Osa T, Peters J, Neumann G, Giordano PR (2017) A learning-based shared control architecture for interactive task execution. In: Proc. IEEE Int. Conf. Robot. Autom. (ICRA), pp 329–335

[11] Rosenberg LB (1993) Virtual fixtures: perceptual tools for telerobotic manipulation. In: Proc. IEEE Virtual Reality Annual International Symposium, pp 76–82

[12] Raiola G, Lamy X, Stulp F (2015) Co-manipulation with multiple probabilistic virtual guides. In: Proc. IEEE/RSJ Int. Conf. Intell. Robot. Syst. (IROS), Hamburg, Germany, pp 7–13

[13] Ewerton M, Maeda G, Koert D, Kolev Z, Takahashi M, Peters J (2019) Reinforcement learning of trajectory distributions: applications in assisted teleoperation and motion planning. In: Proc. IEEE/RSJ Int. Conf. Intell. Robot. Syst. (IROS), pp 4294–4300

[14] Abi-Farraj F, Pedemonte N, Robuffo Giordano P (2016) A visual-based shared control architecture for remote telemanipulation. In: Proc. IEEE/RSJ Int. Conf. Intell. Robot. Syst. (IROS), pp 4266–4273

[15] Havoutis I, Calinon S (2017) Supervisory teleoperation with online learning and optimal control. In: Proc. IEEE Int. Conf. Robot. Autom. (ICRA), IEEE 1534–1540

[16] Havoutis I, Calinon S (2019). Learning from demonstration for semi-autonomous teleoperation. Auton Robots.

[17] Gao X, Silvério J, Pignat E, Calinon S, Li M, Xiao X (2021). Motion mappings for continuous bilateral teleoperation. IEEE Robot Automat Lett.

[18] Perrin N, Schlehuber-Caissier P (2016). Fast diffeomorphic matching to learn globally asymptotically stable nonlinear dynamical systems. Syst Control Lett.

[19] Conti F, Khatib O (2005) Spanning large workspaces using small haptic devices. In: Proc. World Haptics Conference (WHC), pp 183–188

[20] Ratliff ND, Issac J, Kappler D, Birchfield S, Fox D (2018) Riemannian motion policies. arXiv preprint arXiv:1801.02854

